# The Danish prehospital emergency healthcare system and research possibilities

**DOI:** 10.1186/s13049-019-0676-5

**Published:** 2019-11-04

**Authors:** Tim Alex Lindskou, Søren Mikkelsen, Erika Frischknecht Christensen, Poul Anders Hansen, Gitte Jørgensen, Ole Mazur Hendriksen, Hans Kirkegaard, Peter Anthony Berlac, Morten Breinholt Søvsø

**Affiliations:** 1 Centre for Prehospital and Emergency Research, Department of Clinical Medicine, Aalborg University, Søndre Skovvej 15, 9000 Aalborg, Denmark; 2 Department of Regional Health Research, University of Southern Denmark, J. B. Winsløws Vej 19, 5000 Odense, Denmark; 3 Emergency Medical Services, North Denmark Region, Hjulmagervej 20, 9000 Aalborg, Denmark; 4 The Prehospital Organisation, The Region of Southern Denmark, Damhaven 12, 7100 Vejle, Denmark; 5 Prehospital Emergency Medical Services, Region Zealand, Fælledvej 1, 4200 Slagelse, Denmark; 6 Department of Research and Development, Emergency Medical Services, Olof Palmes Alle 34, 8200 Aarhus N, Central Denmark Region Denmark; 7 Copenhagen Emergency Medical Services, Telegrafvej 5, 2. stairway, 3. floor, 2750 Ballerup, Denmark; 8 Centre for Prehospital and Emergency Research, Department of Clinical Medicine, Aalborg University, Søndre Skovvej 15, 9000 Aalborg, Denmark

**Keywords:** Prehospital, EMS, Denmark, Out-of-hours, Emergency number, General practitioner

## Abstract

The emergency medical healthcare system outside hospital varies greatly across the globe - even within the western world. Within the last ten years, the demand for emergency medical service systems has increased, and the Danish emergency medical service system has undergone major changes.

Therefore, we aimed to provide an updated description of the current Danish prehospital medical healthcare system.

Since 2007, Denmark has been divided into five regions each responsible for health services, including the prehospital services. Each region may contract their own ambulance service providers. The Danish emergency medical services in general include ambulances, rapid response vehicles, mobile emergency care units and helicopter emergency medical services. All calls to the national emergency number, 1-1-2, are answered by the police, or the Copenhagen fire brigade, and since 2011 forwarded to an Emergency Medical Coordination Centre when the call relates to medical issues. At the Emergency Medical Coordination Centre, healthcare personnel assess the situation guided by the Danish Index for Emergency Care and determine the level of urgency of the situation, while technical personnel dispatch the appropriate medical emergency vehicles. In Denmark, all healthcare services, including emergency medical services are publicly funded and free of charge. In addition to emergency calls, other medical services are available for less urgent health problems around the clock. Prehospital personnel have since 2015 utilized a nationwide electronic prehospital medical record. The use of this prehospital medical record combined with Denmark’s extensive registries, linkable by the unique civil registration number, enables new and unique possibilities to do high quality prehospital research, with complete patient follow-up.

## Background

Prehospital healthcare, or emergency medical services (EMS), varies greatly across the globe, even within western countries, and is under a continuous development. Several previous studies have elucidated the systems and setups in different countries [1–7]. However, the most recent description of the Danish prehospital healthcare system, was a publication from 2004 [8]. Within the last ten years, the Danish prehospital system has undergone major changes with the implementation of Emergency Medical Coordination Centres (EMCC) (2011), a national helicopter emergency medical service (HEMS) (2014), and a nationwide electronic prehospital medical record (2015). At the same time, the demand for EMS has increased - not only in Denmark [9–11]. Consequently, the interest in prehospital research is rapidly growing. To aid researchers and others with interest in the field, we therefore aimed to provide an updated description of the current Danish prehospital healthcare system and the research potential within this field, with special focus on the EMS.

### Setting

**Fig. 1:**
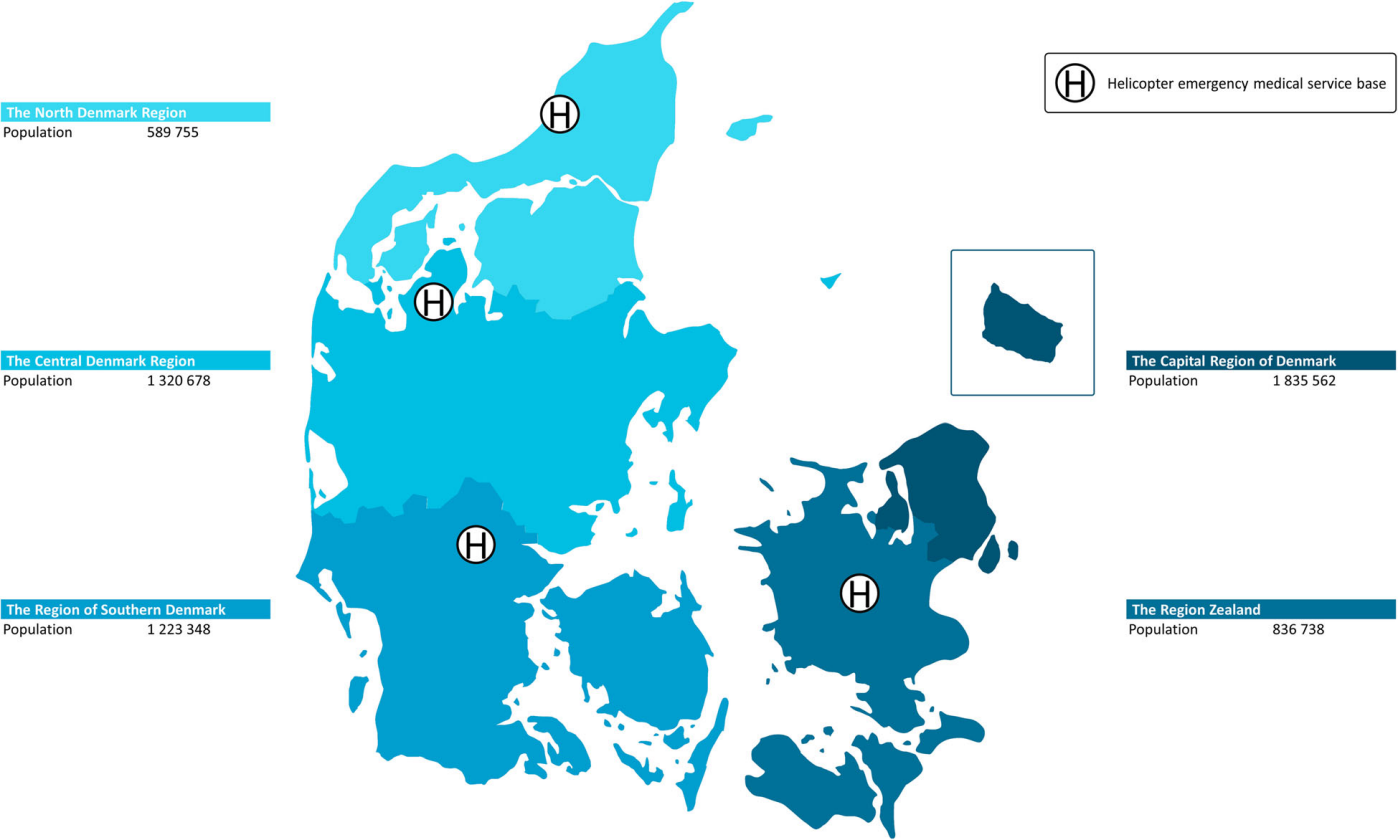
Denmark. The five Danish regions with population as of the first quarter of 2019

Denmark is located in northern Europe with a population of approximately 5.8 million inhabitants as of 2019 and it covers 43,094 km^2^ (16,639 mile^2^).The country has since 2007 been divided into five regions; North Denmark Region, Central Denmark Region, Region of Southern Denmark, Region Zealand, and Capital Region of Denmark (see Fig. 1). Within the framework of Danish legislation, each region is responsible for healthcare services such as general practitioners, EMS and hospitals, whereas the municipalities are responsible for other aspects of community-based healthcare. The population, population growth, and rural/urban areas vary between each region. As a publicly funded system, the general Danish healthcare services are available to all Danish citizens. The prehospital system and the in-hospital emergency care system are available to non-citizens as well. Each Danish citizen has a unique civil personal registration number [12, 13]. This is used by all public authorities as a unique identification. Hence, contacts to healthcare of any kind are registered using the citizens’ civil personal registration number, enabling linkage between several regional and national registers.

### Prehospital system

Each of Denmark’s five regions has a prehospital organization responsible for the EMS. The national *executive order on health preparedness planning* states that the purpose of EMS care is to:

*“Save lives, improve health prospects, reduce pain and other symptoms, shorten the overall duration of illness, provide care and safety.*” [14]

Ambulance services may be operated by the region or contracted from private companies. Private companies must uphold requirements determined by each region. The requirements for ambulances and ambulance personnel are however nationally governed by law [15–17]. There are two levels of ambulance personnel with increasing competences; paramedic, and paramedic with special competencies. Paramedics with special competencies can be further specialised for supplemental competencies, defined by each region.

Since July 1st 2019 ambulance personnel have been authorised as other healthcare professionals, by the Danish Patient Safety Authority [18]. The ambulance personnel all work by delegation from a physician, and the regional prehospital medical director has the overall responsibility for the prehospital treatment in the region.

The basic prehospital resource is an ambulance manned with two professionals, one of which is at paramedic level or higher. Paramedics with special competences, both independently in rapid response vehicles, and together with prehospital anaesthesiologists in mobile emergency care units, are available as a supplemental resource. Due to the diverse ambulance service providers, and regional aims, a different selection of resources are available in each region.

Denmark has a national HEMS, which was officially entered into service in 2014 [19, 20]. It consists of four helicopters manned by a pilot, an anaesthesiologist, and a specially trained paramedic [19]. The HEMS provide 24-h coverage for the entire country, and the helicopters are capable of transporting a single patient.

Although a part of the Danish Defence, the Joint Rescue Coordination Centre Denmark which provides search and rescue, may be called upon to assist in emergency medical situations in Denmark [21].

Finally, several volunteer layperson first aiders are present in the regions. These include, among others, trained volunteers equipped with automatic electronic defibrillators who receive alerts if there is a medical emergency in their vicinity [22, 23]. Application and text-message based alert systems are also present for volunteers. Automatic electronic defibrillators are widely available in the entire country for public use [24].

Since 2015, all Danish medical emergency vehicles, with the exception of HEMS, use an electronic prehospital medical record. The medical record is located on a specially designed tablet and is identical in the entire country. Ambulance personnel enter data, and forward information to hospitals through the electronic medical record.

### Prehospital pathway

The Danish emergency number, 1–1-2, covers all emergencies: police, fires and emergency medical situations and is solely intended for emergencies where urgent assistance is required [25]. The number can be used free of charge from all telephones. All 1-1-2 calls are initially answered by the police (except for parts of the Capital Region of Denmark, where the Copenhagen fire brigade answer the calls). The police assess the call and locate the site of the incident. Since 2011, if a call is of a medical nature, the police then forward the call to a regional EMCC [26]. It is not possible to call the EMCC directly in Denmark.

At the EMCC, the call is received by healthcare professionals, these include nurses and paramedics. Depending on the region, a physician may also be physically present (otherwise available by telephone). The healthcare professionals assess the situation using the criteria-based dispatch decision support tool, the Danish Index for Emergency Care (Danish Index) [27, 28]. This is divided into 37 criteria corresponding to clinical signs, symptoms or incidents. This tool support the healthcare professionals in deciding the response, according to the level of urgency (see Table 1), including the possibility to not send any response (urgency level E). The Danish Index is used in all Danish regions.

**Table 1:** Danish index for emergency care

A - Acute	Acute situation assessed as potentially life threatening
B - Urgent	Urgent situation but not assessed as acute life threatening
C - Scheduled	Non-acute situations, but with need for observation and treatment in ambulance
D - Supine transport	Transportation while lying down, without need for observation or treatment
E - Other services	Other help such as taxi, directing to other healthcare services, advice, etc.

The urgency levels used in the Danish Index for Emergency Care

### The urgency levels used in the Danish index for emergency care

Technical dispatch personnel are also present in the EMCC. They are responsible for logistics and dispatching the appropriate medical emergency vehicles corresponding to the level of urgency assessed by the healthcare professionals.

When the ambulance is dispatched, the ambulance personnel receive information on the patient and the situation. They may also receive digital notes from the healthcare professional at the EMCC who may still be in contact with the caller, thereby relaying new information.

The EMCC also handles the non-acute ambulance runs and transportations. General practitioners (GP), hospital wards and others can contact the EMCC for both acute and non-acute transports.

As a criteria based decision support tool, the Danish Index is intended to determine the urgency of the medical problem, rather than having the healthcare personnel assign a diagnosis over the telephone. Ambulance personnel record their prehospital assessment of the patient and continuously perform treatment and record observations during transport. Diagnoses are only assigned to the patient if a physician is present on scene (a select number of International Statistical Classification of Diseases and Related Health Problems 10th Revision (ICD-10) diagnoses are available for the physician in the prehospital medical record) [29]. All patients with hospital contact are required to receive a diagnosis within ICD-10.

### Other ways of accessing the emergency healthcare systems

**Fig. 2:**
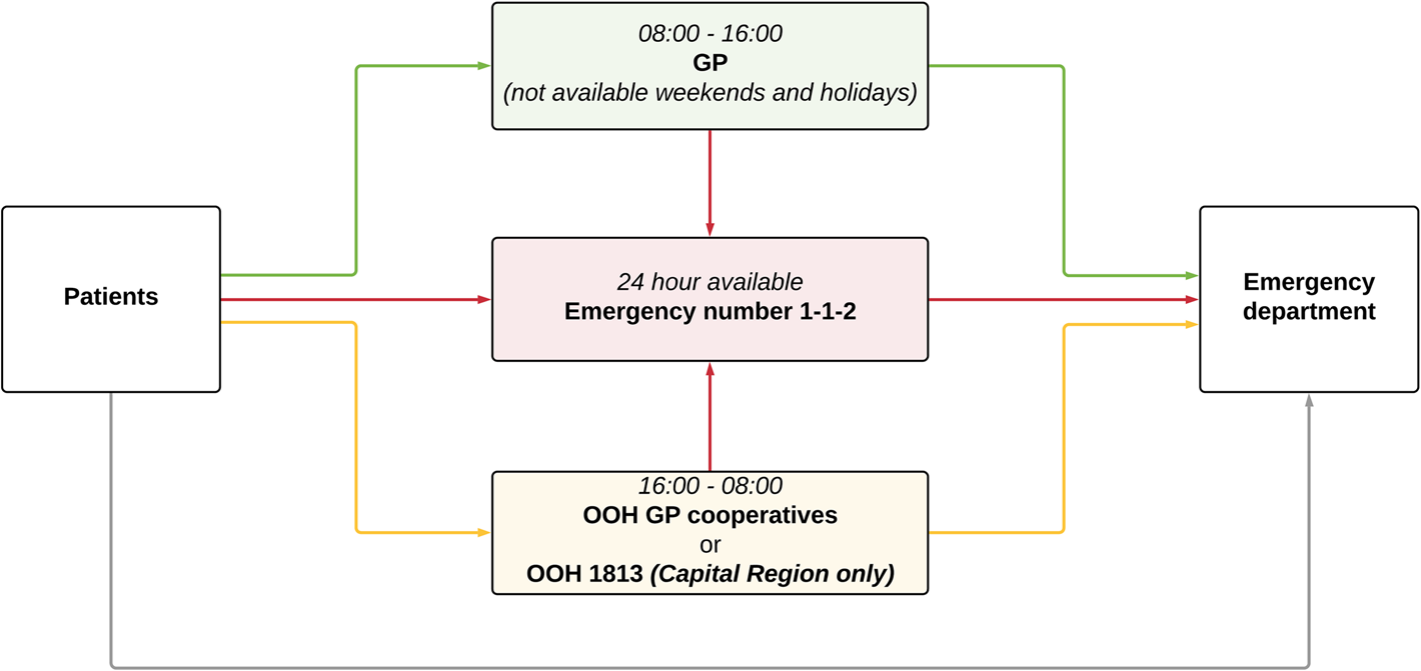
Medical service accesses. The options available for citizens in need of medical services. GP, General practitioner. OOH GP cooperatives, Out-of-hours general practitioner cooperatives. OOH 1813, Out-of-hours Medical Helpline 1813 in the Capital Region

Danish citizens have several options when in need of medical services. All of them are free of charge through the publicly funded healthcare system. (Fig. 2)

#### General practitioner

GPs are available during normal daytime working hours. Patients can, among other options, receive face-to-face, telephone or e-mail consultations, and renew prescriptions for medications [30]. The GPs may refer the patient to other specialists, emergency departments, or other hospital departments. The GP may request ambulances by calling the EMCC.

#### Out-of-hours general practitioner cooperatives

Outside of the normal working hours, patients can contact one of the GP-cooperatives for medical assistance [25]. The GP-cooperatives are operated by GPs who assess the patient by phone, deciding whether the patient is in need of telephone advice only, a consultation/home visit, or a direct referral to hospital. The intended use for the GP-cooperatives is less urgent health problems that cannot wait until the patient’s own GP is available. This can include sudden illness or deterioration of health and minor to moderate injuries.

#### Out-of-hours Medical Helpline 1813

The Capital Region of Denmark has the Medical Helpline 1813 (1813). It is operated by nurses and physicians that use a decision support tool to help determine the appropriate aid. This may include advice over the telephone, referral to consultation at a hospital, home visit (performed by physicians from 1813), or direct hospital referral for admission [31].

#### Emergency departments

Access to the Danish emergency departments requires prior referral by the healthcare system. This may be obtained by either calling the GP-cooperatives, 1813, or the emergency number 1-1-2 [32]. However, citizens can still show up at the emergency departments on their own accord but are prompted to call one of the out-of-hours healthcare services.

#### National acute healthcare number 1-1-3

The Danish Regions have decided to establish a national number, 1-1-3, as access to the acute healthcare system. The number is not yet implemented, but may function as the out-of-hours GPs and 1813, providing advice over the phone, consultations/home visits, or direct referrals to a hospital [33]. The number of prehospital calls per year can be seen in Table 2.

**Table 2:** Prehospital and out-of-hours calls per 1000 capita

Tasks	North Denmark Region	Central Denmark Region	Region of Southern Denmark	Region Zeeland	Capital Region of Denmark
Emergency number calls redirected to EMCC	57	44	45	64	62
Level of urgency: A - Acute	31	20	27	38	23
Out-of-hours general practitioner / 1813^a^	496	518	564	638	433

The number of prehospital and out-of-hours calls per 1000 capita, for each of Denmark’s five regions. Includes numbers from 2016, the most recent year information from both groups were available [34, 35]. EMCC, Emergency Medical Coordination Centre. 1813, Out-of-hours Medical Helpline in the Capital Region

^a^Estimated numbers. Data are currently only available from an evaluation report including numbers collected with different procedures and time intervals, comparisons should be made with caution

### Emergency patient care pathway in prehospital research

The extensive Danish registries and the unique civil registration number allows for conducting prehospital research of high quality following the patient from emergency medical call to hospital discharge. Furthermore, the same electronic prehospital medical record is used throughout the country, and patients are all assessed using the Danish Index.

Since 1994, when a patient is admitted to a hospital, it has been a requirement that the patient receives a diagnosis within Danish SKS Classifications (corresponding to the ICD-10 with additional Danish classifications) [29, 36–39]. These diagnoses and other details in relation to hospitalisation are available from the National Patient Registry. A vast amount of other relevant registries exist [20, 40], where the majority allows for linkage through use of the civil registration number. Among others, this enables the possibility to monitor the prehospital treatment, diagnostic pattern, and outcome (e.g. mortality or return to work). As such it is possible to include the entire patient pathway from the emergency medical call to hospital discharge, and perform complete follow-up of prehospital patients in Denmark.

The Danish Clinical Registries manages, improves, and aids the utilisation of National Clinical Quality Databases [41]. One of these clinical quality databases is the Danish Quality Database for Prehospital Emergency Medical Services, which aims to establish national quality assessment and improvement through monitoring the prehospital care in Denmark [26, 42]. Historically, this database has primarily held time intervals and only few clinical and patient outcome measurements, due to data availability. However, the nationwide electronic prehospital medical record now also provides clinical data, which enables follow-up by linkage to in-hospital medical records and registries. Each of the regional prehospital organisations has access to data on their EMS patients. However, prehospital data is a mix of calls, ambulances and patients. In order to define the “EMS patient care pathway” the regional EMS medical directors and The Danish Clinical Registries initiated a project in 2018. The aim is to establish a common definition of key variables representing the prehospital patient care pathway, and the first version is planned to be implemented by the end of 2019. This will support the prehospital organisations at an administrative level, aid the quality improvement programs, and enable research on both regional and national level.

Linkage between registries are facilitated by the use of the civil registration number, however as a part of the European Union, data protection in Denmark is regulated by the General Data Protection Regulation, supplemented by specific Danish regulations and the Danish Executive Order of the Health Act which specifies when patient medical information may be accessed for research [43–45]. The National Committee on Health Research Ethics, and/or the Danish Patient Safety Authority can approve research projects for data access, when patient consent is not possible. Research involving medical equipment or medication require approval from both a regional Committee on Health Research Ethics and the Danish Medicines Agency [46–48].

## Conclusions

The Danish prehospital system encompasses the national emergency call 1-1-2 answered by the police and forwarded to an EMCC, and the ambulance services. Supplementing this system, other medical services are available for less urgent health problems around the clock, in all of Denmark.

The prehospital system has undergone major changes since last described by *Langhelle* et al in 2004, prominently the employment of healthcare professionals in the EMCCs, implementation of the Danish Index, and use of the electronic prehospital medical record [8, 28]. Furthermore, the prehospital system has been supplemented by the national HEMS in 2014.

Along with the extensive Danish registries, the nation-wide prehospital patient record has enabled high quality research and quality assurance projects. Thus, knowledge of the entire prehospital patient population can now be obtained. This opens for the possibility to achieve knowledge of the entire prehospital patient population and related outcomes.

This description of the current Danish prehospital healthcare setup may aid international researchers in better understanding the Danish prehospital setup.

We believe that multicentre studies and population-based studies should be prioritized in future prehospital research. Updated descriptions such as the present study will enable relevant comparisons of prehospital systems internationally. Therefore, we encourage others to likewise describe their current prehospital setups as well.

## Data Availability

Not applicable

## Abbreviations

1813: Medical Helpline 1813
Danish Index: Danish Index for Emergency Care
EMCC: Emergency Medical Coordination Centre
EMS: Emergency medical services
GP: General practitioner
HEMS: Helicopter emergency medical service
